# Effectiveness of Cultural Adaptations of Interventions Aimed at Smoking Cessation, Diet, and/or Physical Activity in Ethnic Minorities. A Systematic Review

**DOI:** 10.1371/journal.pone.0073373

**Published:** 2013-10-07

**Authors:** Vera Nierkens, Marieke A. Hartman, Mary Nicolaou, Charlotte Vissenberg, Erik J. A. J. Beune, Karen Hosper, Irene G. van Valkengoed, Karien Stronks

**Affiliations:** 1 Department of Public Health, Academic Medical Center – University of Amsterdam, Amsterdam, The Netherlands; 2 Department of Epidemiology, Documentation and Health Promotion, Public Health Service of Amsterdam, Amsterdam, The Netherlands; Consejo Superior de Investigaciones Cientifics, Spain

## Abstract

**Background:**

The importance of cultural adaptations in behavioral interventions targeting ethnic minorities in high-income societies is widely recognized. Little is known, however, about the effectiveness of specific cultural adaptations in such interventions.

**Aim:**

To systematically review the effectiveness of specific cultural adaptations in interventions that target smoking cessation, diet, and/or physical activity and to explore features of such adaptations that may account for their effectiveness.

**Methods:**

Systematic review using MEDLINE, PsycINFO, Embase, and the Cochrane Central Register of Controlled Trials registers (1997–2009). Inclusion criteria: a) effectiveness study of a lifestyle intervention targeted to ethnic minority populations living in a high income society; b) interventions included cultural adaptations and a control group that was exposed to the intervention without the cultural adaptation under study; c) primary outcome measures included smoking cessation, diet, or physical activity.

**Results:**

Out of 44904 hits, we identified 17 studies, all conducted in the United States. In five studies, specific cultural adaptations had a statistically significant effect on primary outcomes. The remaining studies showed no significant effects on primary outcomes, but some presented trends favorable for cultural adaptations. We observed that interventions incorporating a package of cultural adaptations, cultural adaptations that implied higher intensity and those incorporating family values were more likely to report statistically significant effects. Adaptations in smoking cessation interventions seem to be more effective than adaptations in interventions aimed at diet and physical activity.

**Conclusion:**

This review indicates that culturally targeted behavioral interventions may be more effective if cultural adaptations are implemented as a package of adaptations, the adaptation includes family level, and where the adaptation results in a higher intensity of the intervention. More systematic experiments are needed in which the aim is to gain insight in the best mix of cultural adaptations among diverse populations in various settings, particularly outside the US.

## Introduction

There is a high prevalence of chronic diseases among ethnic minorities in high-income societies. For example, hypertension and diabetes are highly prevalent among populations of African and South-Asian origin in the United States and Europe, particularly compared with populations of European origin in those regions [1], [2]. Important preventable risk factors for these diseases are health-related behaviors that include smoking, diet and physical activity (PA). Hence, interventions to promote healthier behaviors are crucial to a reduction in these diseases. Often, however, interventions targeted to the general population do not reach ethnic minorities. In addition, there are indications that such interventions have limited effects on health behavior [3]–[5].

Increasing the cultural sensitivity of lifestyle interventions is generally expected to enhance their appropriateness and effectiveness [3]. Cultural sensitivity can be described as the extent to which a target population's ethnic and/or cultural characteristics, experiences, norms, values, behavioral patterns, and beliefs as well as relevant historical, environmental, and social forces are incorporated into the design, delivery, and evaluation of targeted health promotion materials and programs [6]. This can be done through cultural adaptations such as matching materials to group characteristics or targeting cultural values of the population [6].

Increasing numbers of studies and reviews have examined the effectiveness of a broad range of culturally adapted interventions targeted at a variety of diseases (i.e. diabetes, asthma, or stroke) or health-related behaviors such as physical activity (PA) [7]–[11]. However, none of them make clear whether the reported effectiveness can be attributed to particular cultural adaptations. More importantly, in most of the studies included in previous reviews, different types of interventions were used for the control and intervention groups. For example, a culturally adapted 12-week group intervention compared with a control group that received a leaflet only. The differences in study arms of such studies do not allow us to draw firm conclusions about the effectiveness of the cultural adaptations.

Instead, a study design in which the only variation between the intervention and control group is the specific cultural adaptation that will be studied on effectiveness, is precisely what is needed to gain more insight into the effectiveness of specific cultural adaptations, and to determine which adaptations are really necessary and which are not.

This paper aims to fill this gap by systematically reviewing randomized and non-randomized studies that evaluate the effectiveness of health interventions targeted to ethnic minorities and focusing on smoking cessation, diet and/or PA. We were interested in evaluating studies in which the intervention *without* a particular cultural adaptation is compared with the same intervention *with* a particular cultural adaptation. Our specific objectives were:

1. to systematically review the effectiveness of cultural adaptations in interventions that targeted smoking cessation, diet and/or PA among ethnic minorities in high income societies, in terms of changes in the abovementioned behaviors.
2. to explore those features of cultural adaptations that may account for their effectiveness.

## Methods

We conducted a systematic review of the effectiveness of culturally sensitive interventions that targeted smoking cessation, diet and/or PA among adults from ethnic minorities in high income societies. We based our review methodology on the Guidelines for Systematic Reviews of Health Promotion and Public Health Interventions [12]. The protocol has not been published or registered but is detailed below.

The protocol included the aim, the search strategy (Table 1) and the selection procedure. For the selection procedure, we developed a registration sheet in which the studies were registered and in which the reason for selection or non-selection was registered.

**Table 1:** Search strategies.

	Embase	Medline	Psychinfo	Cochrane CT
**Basic search**	1. exp “ethnic, racial and religious groups”/or exp “ethnic or racial aspects”/or exp minority group/or racial group*. ti,ab. or minority group*. ti,ab. or ethnic*. ti,ab. or Moroc*. ti,ab. or Turk*. ti,ab. or Surinam*. ti,ab. or Antill*. ti,ab. or emigrant*. ti,ab. or immigrant*. ti,ab. or transient*. ti,ab. or migrant*. ti,ab. or exp eastern hemisphere/or exp oceanic regions/or exp western hemisphere/or cultur*. ti,ab.	1. exp “emigrants and immigrants”/or exp population groups/or exp “transients and migrants”/or emigrant. ti,ab. or immigrant. ti,ab. or transient*. ti,ab. or migrant. ti,ab. or exp Minority Groups/or minority. ti,ab. or exp geographic locations/or Moroc*. ti,ab. or Turk*. ti,ab. or surinam*. ti,ab. or Antill*. ti,ab. or exp culture/or cultur*. ti,ab.	1. exp “racial and ethnic groups”/or exp minority groups/or migrant*. ti,ab. or exp minority groups/or surinam*. ti,ab. or Turk*. ti,ab. or Moroc*. ti,ab. or Antill*. ti,ab. or exp “culture (anthropological)”/or exp diversity/or exp socio-cultural factors/or exp minority groups/or exp cultural sensitivity/or exp multiculturalism/	#1 MeSH descriptor Emigrants and Immigrants explode all trees; #2 MeSH descriptor Population Groups explode all trees; #3 MeSH descriptor Transients and Migrants explode all trees; #4 (emigrant): ti,ab,kw;#5 (immigrant): ti,ab,kw; #6 (transient*): ti,ab,kw; #7 (migrant): ti,ab,kw; #8 MeSH descriptor Minority Groups explode all trees;#9 (minority): ti,ab,kw; #10 MeSH descriptor Geographic Locations explode all trees; #11 (Moroc*): ti,ab,kw; #12 (Turk*): ti,ab,kw; #13 (surinam*): ti,ab,kw; #14 (Antill*): ti,ab,kw; #15 MeSH descriptor Culture explode all trees; #16 (cultur*): ti,ab,kw; #17 (#1 OR #2 OR #3 OR #4 OR #5 OR #6 OR #7 OR #8 OR #9 OR #10 OR #11 OR #12 OR #13 OR #14 OR 15 OR #16); #18 MeSH descriptor Health Education explode all trees; #19 (Health Education): ti,ab,kw; #20 (Health Promotion): ti,ab,kw; #21 MeSH descriptor Health Promotion explode all trees; #22 MeSH descriptor Preventive Health Services explode all trees; #23 (prevent*): ti,ab,kw; #24 (interven*): ti,ab,kw; #25 (#18 OR #19 OR #20 OR #21 OR #22 OR #23 OR #24); #26 (#17 AND #25); #27 (#26), from 1997 to 2009
	2. health education. ti,ab. or exp health education/or health promotion. ti,ab. or exp disease control/or preven*. ti,ab. or interven*. ti,ab	2. exp Health Education/or health education. ti,ab. or exp Health Promotion/or health promotion. ti,ab. or exp Preventive Health Services/or interven*. ti,ab. or prevent*. ti,ab	2. health promotion. ti,ab. or exp Health Promotion/or health education. ti,ab. or exp Health Education/or exp intervention/or exp prevention/or preven*. ti,ab. or interven*. ti,ab.	
**Behavior specific supplement**	**Smoking**	**Smoking**	**Smoking**	**Smoking**
	3. exp “smoking and smoking related phenomena”/or exp smoking cessation/or exp nicotine replacement therapy/or exp smoking cessation program/or smok*. ti,ab. or tobacco. ti,ab	3. exp Smoking/or smok*. ti,ab. or exp Smoking Cessation/or exp “Tobacco Use Cessation”/or tobacco. ti,ab.	3. smoking cessation. mp. or exp smoking cessation/	#28 MeSH descriptor Tobacco Use Cessation explode all trees; #29 (smoking cessation): ti,ab,kw; #30 (#28 OR #29); #31 (#27 AND #30)
	**Physical activity**	**Physical activity**	**Physical activity**	**Physical activity**
	3. (exp Exercise/or fitness/or exp Physical Education/or exp Sport/or exp Dancing/or exp Kinesiotherapy/or (physical$ adj5 (fit$ or train$ or activ$ or endur$)). tw. or ((exercis$ adj5 (train$ or physical$ or activ$)) or sport$ or walk$ or bicycle$ or (exercise$ adj aerobic$) or ((“lifestyle” or life-style) adj5 activ$) or ((“lifestyle” or life-style) adj5 physical$)). tw.)	3. exp Physical Exertion/or Physical Fitness/or exp “Physical Education and Training”/or exp Sports/or exp Dancing/or exp Exercise Therapy/or (physical$ adj5 (fit$ or train$ or activ$ or endur$)). tw. or (exercis$ adj5 (train$ or physical$ or activ$)). tw. or sport$. tw. or walk$. tw. or bicycle$. tw. or (exercise$ adj aerobic$). tw. or ((“lifestyle” or life-style) adj5 activ$). tw. or ((“lifestyle” or life-style) adj5 physical$). tw.	3. exp Exercise/or exp Physical Fitness/or exp Physical Education/or exp Sports/or exp dance/or exp Movement Therapy/or ((physical$ adj5 (fit$ or train$ or activ$ or endur$)) or ((exercis$ adj5 (train$ or physical$ or activ$)) or sport$ or walk$ or bicycle$ or (exercise$ adj aerobic$) or ((“lifestyle” or life-style) adj5 activ$) or ((“lifestyle” or life-style) adj5 physical$))). tw.	#28 MeSH descriptor **Physical Exertion** explode all trees; #29MeSH descriptor **Physical Education and Training** explode all trees; #30 MeSH descriptor **Dancing** explode all trees; #31 (physical*): ti,ab,kw; #32 (endur*): ti,ab,kw; #33 (exercis*): ti,ab,kw; #34 (sport*): ti,ab,kw; #35 (walk*): ti,ab,kw; #36 (bicycle*): ti,ab,kw; #37 (aerobic*): ti,ab,kw; #38 (lifestyle): ti,ab,kw; #39(life-style): ti,ab,kw; #40 (#**28** OR #**29** OR #**30** OR #**31** OR #**32** OR #**33** OR #**34** OR #**35** OR #**36** OR #**37** OR #**38** OR #**39**); #41 (#**27** AND #**40**)
	**Diet**	**Diet**	**Diet**	**Diet**
	3. nutrition*. ti,ab. or exp Body Weight/or exp Weight Reduction/or weight. ti,ab. or exp Diet Therapy/or exp Diet/or diet*. ti,ab. or diabet*. ti,ab. or exp Food Intake/or exp Nutritional Status/or exp Food Preference/	3. nutrition*. ti,ab. or exp Body Weight/or exp Weight Loss/or weight. ti,ab. or exp Diet Therapy/or exp Diet/or diet*. ti,ab. or diabet*. ti,ab. or “Food Habits”/or “Nutritional Status”/or “Food Preferences”/	3. nutrition*. ti,ab. or exp Body Weight/or exp Weight Loss/or weight. ti,ab. or exp Diets/or diet*. ti,ab. or diabet*. ti,ab. or exp FoodIntake/or exp Nutrition/or exp Food Preferences/	#28 (nutrition): ti,ab,kw; #29 MeSH descriptor; #30 **Weight** Loss explode all trees; #31weight loss: ti,ab,kw; #32MeSH descriptor **Diet Therapy** explode all trees; #33 MeSH descriptor Diet explode all trees; #34 (diet): ti,ab,kw; #35 MeSH descriptor **Food Habits** explode all trees; #36 MeSH descriptor **Nutritional Status** explode all trees; #37 MeSH descriptor **Overweight** explode all trees; #38 (#28 OR #29 OR #30 OR #31 OR #32 OR #33 OR #34 OR #35 OR #36); (#27 AND #37)
	4. 3 and 1 and 2	4. 3 and 1 and 2	4. 3 and 1 and 2	Time limit included in basic search
	5. limit 4 to year = ′′1997–2009′′	5. limit 4 to year = ′′1997–2009′′	5. limit 4 to year = ′′1997–2009′′	

We developed, tested, adjusted, and retested the search strategy to ensure it was as sensitive as possible. We searched for controlled studies with no language restrictions in MEDLINE, PsycINFO, Embase (all from January 1997-September 2009), and the Cochrane Clinical Trial Database (January 1997-April 2010). We choose to include papers that were written in the context of increased refinement of culturally sensitive interventions as defined by Resnicow in his landmark paper in 1999 [6]. The year 1997 was chosen as the starting point to minimize the chance for missing important studies.

To ensure the consistency of the search strategy across the three different domains (smoking, diet and PA), the strategy consisted of two parts: a basic search strategy aimed at finding intervention studies among ethnic minority populations, and a behavior-specific supplement aimed at the three behaviors. Both MeSH terms and free-text words were used (Table 1).

Subsequently, the publications from each database were cross-checked manually and duplicates were removed. At least two reviewers per lifestyle domain (smoking cessation, diet and PA) screened the titles and abstracts of the identified publications and excluded all publications that clearly did not meet our inclusion criteria. For all the other articles, full texts were obtained and two reviewers independently assessed eligibility. If there was uncertainty about inclusion, consensus was achieved by discussion.

Criteria for inclusion in our review:

- The study described the effect evaluation of a lifestyle intervention culturally adapted to a specific adult, ethnic minority population living in a high-income society.
- The study included a control group (randomized or non-randomized) that was exposed to the basic intervention without one or more cultural adaptations that were evaluated on their effectiveness in this study (i.e. the adaptation under study).
- The outcome measure of interest was behavior change regarding smoking cessation, diet, or PA.

Our inclusion criteria allowed studies in which the control intervention made use of cultural adaptations but in which the intervention group received one or more additional cultural adaptations (i.e., the specific cultural adaptation under study). Studies were excluded if the control and intervention groups seemed to have received different interventions (e.g., a leaflet versus a culturally adapted group intervention), and the authors did not describe the difference between the intervention and control groups as including a difference in use of cultural adaptations.

Two reviewers per lifestyle domain used a pretested form to independently summarize the data, including study characteristics, intervention characteristics, details about the intervention and cultural adaptations, study design, and intervention effects of the included articles. To define ‘cultural adaptations’ we used the definition of Resnicow et al. [6] for surface-structure and deep-structure adaptations. We considered surface-structure adaptations as those adaptations that match materials or messages to observable, superficial characteristics of the target population. For example, interventions that used pictures of the target population. Deep-structure adaptations are those adaptations that address core cultural values or those ethnic, cultural, historical, social or environmental factors that may influence specific health behaviors. For example, interventions address the role of food in hospitality in the culture of the target population.

For the included articles, the Effective Public Health Practice Project (EPHPP) quality assessment tool for quantitative studies was used to assess the individual studies' risk of bias [13]. This tool rates design, selection bias, allocation bias, confounding, blinding, data-collection methods, withdrawals and dropouts (attrition bias), with “strong”, “moderate,” or “weak”. Based on the number of strong, moderate, or weak scores, the study was given an overall score. Studies were rated as strong if there were no weak ratings, as moderate if there was one weak rating, and as weak if there were more than two weak ratings. Each of the two reviewers rated the quality independently. If there was disagreement, the results were discussed and a final decision was made. We e-mailed all authors of the included studies to retrieve missing information, and completed the quality assessment using the information they provided. We assessed whether we could find an association between studies with a statistically significant effect or no effect and the quality of the studies or whether publication bias could affect the results.

We found a large variety in outcome measures, which made a detailed analysis of effect sizes impossible. Therefore, a narrative synthesis was conducted describing the study design, the intervention and cultural adaptations of the effective and non-effective studies, target population, setting, attrition rates, and reported effectiveness of the intervention (based on statistical significance). To provide detailed information about the results of the studies, we present the effect sizes in the tables. Effect sizes were described in terms of differences in mean and percentage, depending on the outcome measures of the studies. To answer research question two, we compared the characteristics of the adaptations for effective with those of non-effective interventions.

## Results

### Study selection

**Figure 1:**
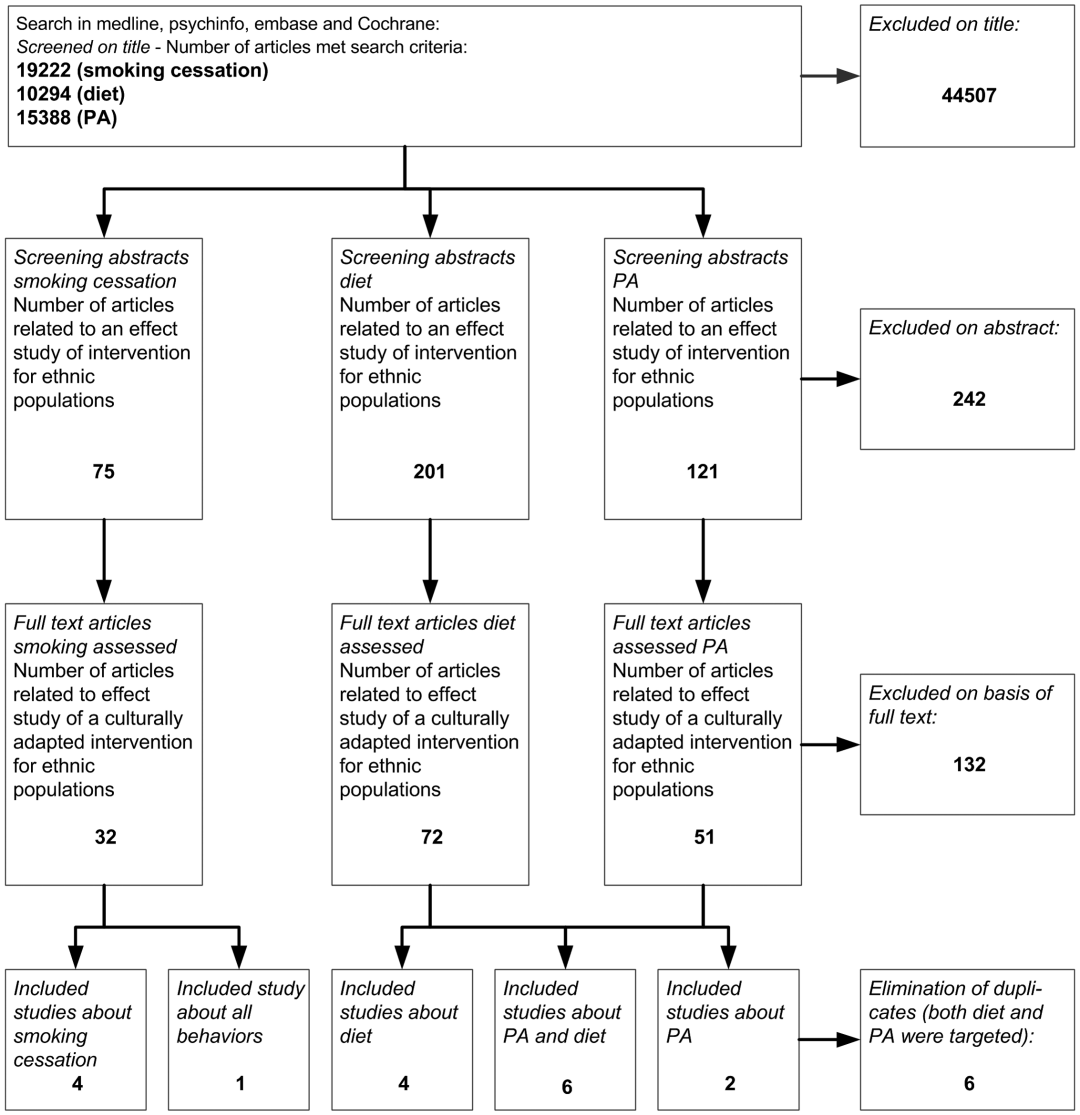
Flowchart of search strategy.

The search across the four databases yielded 19222 publications for smoking cessation, 10294 publications for diet, and 15388 publications for PA (Figure 1). Full texts were obtained for 32 studies on smoking, 72 on diet, and 51 on PA. Of these studies, 132 (27 on smoking, 62 on diet, and 43 on PA) were excluded because the intervention and control groups received different types of interventions. Within the 132 excluded studies was 1 study where the control group spontaneously took over the cultural adaptations, so that it no longer differed from intervention group [14]. We decided to include studies that investigated the effectiveness of additional cultural adaptations to an intervention that already included one or more cultural adaptations. Six studies were found in both the search strategy for diet and the search strategy for PA. One study that was included via the search for smoking cessation also targeted diet and PA. After removal of overlap because of multiple behaviors addressed, this left 17 studies that met all the inclusion criteria: 4 on smoking cessation [15]–[18], 4 on diet [19]–[22], 2 on PA [23], [24], 6 on PA and diet combined [25]–[30], and 1 on all three behaviors [31], [32].

### Description of included studies

All of the included studies were conducted in the United States. Table 2 summarizes their characteristics. Fifteen of the 17 studies were Randomized Controlled Trials (RCT's). Two studies used a quasi-experimental design. Eleven studies were targeted to African Americans [15], [16], [19], [21], [22], [24], [25], [27]–[29], five to a Latino population [17], [18], [20], [26], [30] and one study to Chinese Americans [23]. Response rates varied from 31% [22] to 96.5% [16]. Six studies did not report response rates 21,23–25,28. Follow-up rates were generally higher than 65%.

**Table 2:** Characteristics of the studies included.

Reference	Design	Setting and target population	Characteristics participants				Response	Follow-up rate			Outcome measure	Quality assessment score
			Intervention group	Control group				Intervention group	Control group			
***Smoking cessation***												
Wetter et al. 2007 [17]	RCT	Texas (Houston, San Antonio, El Paso, Rio Grande Valley)	N: 149	N: 148			84%	Week 5: 87%	Week 5: 87%		Self reported 7-day abstinence from smoking after 5 weeks and 12 (from baseline measurement)	Strong
		Latino smokers calling CIS with request for smoking cessation help in Spanish, living in Texas, >18 yrs	Age: 41.4 (SD 11.1)	Age: 40.8 (SD 11.8)				Week 12: 80%	Week 12: 80%			
			Men: 55.7%	Men: 54.7%								
			Yrs of education: 10.9 (4.1)	Yrs of education: 10.8 (3.9)								
			Household income <20,000: 56.6%	Household income <20,000: 54.4%								
Orleans et al. 1998 [16]	RCT	US African Americans ≥18 yrs.	N = 733	N = 689			96,5% agreed to follow-up and study participation	6 months: 36.6%	6 months: 67.8%		Self reported one week abstinence at 6 months and 12 months (from baseline measurement)	Moderate
			For both groups:					12 months: 16%	12 months: 17%			(a higher follow-up rate would have resulted in a rating as strong)
			(88% aged 20 –49					Attrition = 84%	Attrition = 83%			
			Female: 63.3%					No significant differences between both groups				
			High school education or more: 84%									
Woodruff et al. 2002 [18]	RCT	California	N = 132 (longitudinal sample)	N = 150 (longitudinal sample)			Not reported	84.6%	95.5%		Self reported 7 day abstinence, CO validated at post treatment (3 months from baseline)	Moderate
		Latino smokers, who intent to quit	Age: 43 (SD 13.7)	Age: 42 (SD 12.2)								
			Female: 52%	Female: 48%								
			Education (mean): 11–13 yrs	Education (mean): 11–13 yrs								
			Income (mean category): $1100 – $1400	Income (mean category): $1100 – $1400								
Nollen et al. 2007 [15]	RCT	South-Eastern United States	N: 250	N: 250			63.5%	4 weeks: 68%	4 weeks: 65.2%		Self reported 7-day abstinence from smoking, CO validated at 4 weeks and 6 months	Strong
		Self identifying African Americans, >18 yrs, in contemplation stage of change, smoking >10 cigarettes a day, had to weigh more >100 lbs	Mean Age: 42.8 (SD 11.0)	Mean Age: 43.1				6 months: 62.8%	6 months: 68.4%			
			Female: 55,2%	(SD 9.8)								
			Monthly income <$1200: 68.8%	Female: 65,2%								
			< high school diploma: 56.7	Monthly income <$1200: 61.6%								
				< high school diploma: 52.8								
***Diet and physical activity combined***												
Babamoto et al. 2009 [26]	RCT with three arms	Inner-city family health centers in Los Angeles, USA	CHW	Case management		Standard provider	68%	CHW:50% at 6 month	Case management group: 43% at 6 month		PA – exercise at least three times per week	Moderate
		Hispanic men and women 18yrs and older with type 2 diabetes	N = 75	N = 60		N = 54			Standard care: 50% at 6 months		Diet – fruit and vegetable intake, frequency of fatty foods eaten daily at six months from baseline.	
			Age: 51.0	Age: 50.0		Age: 50.0			More females (63%) than males (50%) completed the program.		*Other outcomes*	
			Female: 64%	Female: 52%		Female: 78%					BMI and A1C	
			Education <6^th^ grade: 67%	Education <6^th^ grade: 58%		Education <6^th^ grade: 57%						
			Income <$25,000: 55%	Income <$25,000: 50%		Income <$25,000: 56%						
Keyserling et al. 2002 [29]	RCT with 3 arms	Central North Carolina, USA	N = 67	N = 67		N = 66	219 patients screened, 200 randomised (19 did not complete baseline measures)	6m: 89.5%	6m: 90%	6m: 88%	*Primary outcome:*	Strong
		African-American women aged ≥40 years with	Mean Age: 58.5	Mean Age: 59.2		Mean Age: 59.8		12m: 80.6%		12m: 86.5%	PA – Increase moderate intensity PA to 30 minutes per day.	
		type 2 diabetes, defined as diagnosis of	Mean Yrs education: 11.1	Mean Yrs education:11.0		Mean Yrs education:10.1				12m: 89.4%	Assessed with accelerometer (1 week)	
		diabetes at ≥20 years with no history of ketoacidosis									*Other outcomes:*	
											Diet – decrease total and saturated fat intake and improve control and distribution of carbohydrate intake.	
Ard et al. 2008 [25]	Quasi-experiment	USA, Baltimore (Maryland), Durham (North Carolina), and Baton Rouge (Louisiana)	N: 271	N: 106			NA	91%			*Primary outcome:*	Moderate
		African Americans	Mean Age: 51.6 (SD 9.3)	Mean Age: 51.8 (SD 9.2)							Weight change (in kg)	
		Age ≥25 years	Female: 73.4%	Female: 66.3.%							*Secondary outcomes:*	
		BMI 25–45 kg/m2	Annual household income <$29.9 15.7%	Annual household income<$29.9:8.7%							Adherence to diet recommendations	
		Taking prescription medication for either dyslipidemia or hypertension.	Education:	Education:							Adherence to PA recommendations [accelerometer]	
			- High school or some college: 43.7%	- High school or some college: 37.9%							BMI	
Fitzgibbon et al. 2005 [28]	RCT	USA, Chicago (Illinois)	N: 30	N: 29			NA	76.7%	79.3%		PA: energy expenditure, moderate activity and vigorous activity (kcal/kg per day)	Moderate
		Self-identified as African-American or Black	Mean Age: 47.8 (SD 10.3)	Mean Age: 49.1 (SD 11.6)							Individual's usual fat intake (% energy intake)	
		Female	Income ($, median): 20.500	Income ($, median): 20.500								
		Age ≥21 years;	Work full-time outside home (%): 56.7%	Work full-time outside home (%): 37.9%								
		BMI ≥25 kg/M^2^	Education (years): 13.6 (SD 2.3)	Education (years): 12.9 (SD 2.2)								
Staten et al. 2004 [30]	RCT	USA – Arizona.	Provider counseling, health education and community health worker: n = 67	Provider counseling and health education: n = 73		Provider counseling: n = 77	75% baseline response rate.	67% of eligible participants returned at 12 months			Primary outcomes: Fruit and vegetable intakes	Moderate
		Hispanic women 55yrs and older	Mean Age: 57 yrs	Mean Age: 58 years		Mean Age: 56.7 yrs		Not clear what the response was per group as results only show number of women that returned at 12 months.			% participants who undertook moderate-vigorous PA (min/week) weight,	
			Completed high school: 43%	Completed high school: 32%		Completed high school: 34%					BMI	
			Employed: 40%	Employed 33%		Employed 31%						
Campbell et al. 2004 [27]	RCT	USA, North Carolina12 African-American churches	Combined LHA and TPV: n = 176	Lay Health advisor (LHA) only: n = 123		Tailored print material (TPV) only: n = 159	Participant level:66% (Overall CASRO response rate)	LHA + TPV	LHA 62%	TPV 67%	Fruit and vegetable intake: number of servings per day	Moderate
		All church members aged 18 or older.	Age %:	Age %:		Age %:		77%			Percentage meeting 5-a-day rec.	
			>50 = 49.1	>50 = 41.4		>50 = 47.2					Fat intake: % calories from fat	
			Female: 74.0%	Female: 72.4%		Female: 73.6%					Recreational activity (MET hrs/week)	
			Education	Education		Education					% Meeting PA recommendations	
			<high school 15.9%	<high school 20.8%		<high school 11.9%						
**Diet**												
Kreuter et al. 2005 [21], [34]	RCT	USA, St. Louis	N-BCT + CRT: 288	N-BCT: 311		N-CRT: 309	Not reported	1-month follow-up: 80.6%	1-month follow-up: 87.1%	1-month follow-up: 83.8%	Fruit and Vegetable intake at 1,6 and 18 month follow-up	Strong
		African-American women	Mean Age: 35.4 (SD 11.7)	Mean Age: 35.8 (SD 11.5)		Mean Age 35.4 (SD 11.2)		6-month follow-up: 74.0%	6-month follow-up: 82.0%	6-month follow-up: 76.4%		
			Mean Education (years): 12.4 (SD 1.8)	Mean Education (years): 12.2 (SD 1.9)		Mean Education (years): 12.2 (SD 1.9)		18-month follow-up: 68.7%	18-month follow-up: 73.6%	18-month follow-up: 71.8%		
			Pre-tax household income <$20.000/year: 65.0%	Pre-tax household income <$20.000/year: 71.2%		Pre-tax household income <$20.000/year: 68.0%						
Elder et al. 2005, 2006 [20], [33]	RCT	USA, San Diego	N-Promotora (and tailored print): 120	N-Tailored print (only): 118		Not reported	70%	12-week follow-up: 89.2%	12-week follow-up: 83.9%	12-week follow-up 89.9%	*Primary outcomes:*	Moderate
		Latino women	Age: NA	Age: NA				12-months	12-months	12-months	Percent calories form fat and grams of fiber at 12 weeks, 6 and 12 months	
			Education: 0-6yr: 29.2%	Education: 0-6yr: 30.5%				(M1-M4) attrition rate 23%	(M1-M4) attrition rate 24%	(M1-M4) attrition rate 18%	*Secondary outcomes:*	
			Income ($ per month):≤1.000 or 1001–2000: 60.4%	Income ($ per month): ≤1.000 or 1001–2000: 57.1%							Total energy intake, total fat, saturated fat and carbohydrate intake.	
Campbell et al. 1999 [19]	RCT (Cluster (churches) randomized)	USA, eastern North Carolina	N = 108	N = 109			79% in the main study	1-year follow-up: 82%			Fruit and vegetable consumption at 1 and 2 year follow-up	Moderate
		African Americans	Female: 73.0%	Females 74.4%				2-year follow-up: 75.3% (of the subsample who completed the 1-year follow-up survey)				
			Age:	Age								
			18–37: 25 (20.1%)	18–37: 21 (19.9%)								
			38–50: 29 (28.9%)	38–50: 27 (24.8%)								
			51–65: 28 (28.4%)	51–65: 33 (33.2%)								
			65+: 26 (23.0%)	65+: 28 (22.1%)								
			Education:	Education:								
			< high school: 36 (32.5%)	< high school: 42 (39.5%)								
			Income:	Income:								
			<$20.000/yr: 70 (64.9%)	<$20.000/yr: 67 (63.5%)								
Rescinow et al. 2009 [22]	RCT	USA, Detroit Metro area & Atlanta Metro area	N = 372 and N = 188				31%	3-month follow-up: 82%	3-month follow-up: 87%		Fruit and vegetable consumption at 3-month follow-up	Moderate
		Home environment	Female: 73%									
		Self-identifying as Black or African American, living at least half of one lifetime in the United States	Mean age: 49 yrs									
			≥high-school education: 69%									
			≥$40.000/yr: 60%									
			*At baseline, participants in the two study groups did not differ significantly*									
***Physical activity***												
Chiang et al. 2009 [23]	Pretest and posttest quasi-experimental design	USA, Massachusettes	N = 58		N = 70		N.A.: Convenience and snowball sampling	92%	95%		Duration, intensity, frequency of exercises (exercise promotion outcomes monitor)	Weak
		Chinese churches, community center, and Chinese outpatients clinics	Mean age: 73.8 (SD 5.7)		Mean age: 73.1 (SD 6.5)		Enrolment of 137 subjects with 9 lost cases: 128 completed the study: (93.5%)	(Loss to follow-up 5/63)	(Loss to follow- up 4/74)			
	(non probability sample > convenience sampling)	Older (> = 66 yr), Chinese American immigrants (birth foreign to US) with minimum of 6 continuous months of hypertension	Female: 63.8%		Female: 65.6%							
			Household income		Household income							
			<10,000: 63,8%		<10,000: 65,7%							
			Education:		Education:							
			< High-school 51.7%		< High-school: 50%							
Newton et al. 2004 [24]	RCT	Setting not reported	N: 20		Physician Advise (PA) N:10	Standard Behavioral Exercise Counselling (SB) N:22	N.A	80%	PA	SB	Primary outcome:	Moderate
		Sedentary African-American adults	Mean Age: 45 (SD 7.8)		Mean Age: 47.3 (SD 7.4)	Mean Age: 44 (SD 7.0)	(diverse recruitment strategies used)		100%	77%	In cardio respiratory fitness (VO2 max)	
			Female: (81%)		Female: Not reported	Female: not reported					at 6 months follow -up.	
			Mean Yrs of education: 15.3 (SD 2.2)		Mean Yrs of education: 14 (SD 1.4)	Mean Yrs of education: 14.9 (SD 2.1)					Secondary outcomes:	
			Household income: <24,999: 27.8%		House-hold income: <24,999: 25.0%	House-hold income: <24,999: 23.8%					- Self-reported Physical Activity (7 day physical activity recall)	
											-Self-reported adherence to exercise prescription (Self-monitoring exercise logs)	
***All three behaviors***												
Becker et al. 2005/Cene et al. 2008 [31], [32]	RCT	Baltimore, Maryland, USA	N: 196		N: 167		95.3%	5 years 84.2%	5 years 85%		Self report of any smoking within the past month at 1 year and 5 years from baseline	Strong
		African-American men and women (siblings or probrands) of heart patients who participated in the John Hopkins family heart study/30–59 yrs. (The proband is the first affected family member who seeks medical attention for a genetic disorder)	Age: 47.6 (SD 6.7)		Age: 47.9 (SD 5.7)						Verified with expired CO levels.	
			Yrs of education: 12.5 (SD 2.4)		Yrs of education: 13.0 (SD 2.4)							
			Female: 61%		Female: 66%						LDL-C, BMI, MET	
			Employed: 80%		Employed: 77%						(LDL-C = low-density lipoprotein cholesterol, BMI = body mass index, MET = metabolic equivalent)	

Outcome measures in all of the smoking cessation studies were self-reported abstinence. Three studies validated these outcomes with CO levels [15], [16], [18]. Follow-up time varied between four weeks [15], [17] and five years [31], [32]. Primary outcomes varied widely in studies targeting diet and/or PA. For example, Babamoto et al. [26] measured if participants were physically active at least three times a week, while Keyserling et al. [29] measured the PA increase to 30 minutes per day and Fitzgibbon et al. [28] measured energy expenditure as indicator of PA. Outcome measures of studies targeting diet included fruit and vegetable intake and/or fat intake and in one study percent calories from fat and grams of fiber intake [20]. Each study used a different follow-up time. In the one study that targeted all three health behaviors, outcome measures for diet and PA were BMI, LDL cholesterol, energy expenditure and metabolic equivalent [31], [32].

### Quality assessment

Of the 17 included studies 10 authors responded to our questions on clarifications regarding quality assessment. Subsequently, 5 studies were assessed as strong, 11 as moderate, and 1 as weak (Table 2). Moderate and weak studies mostly had problems with attrition rates or lacked information about response or attrition rates. This implies that there might be a chance of selection bias in these studies, namely the possibility that the intervention had a different effect in that part of the study population that was lost to follow-up. Other problems identified were insufficient blinding, not reporting information about reliability of data collection tools or not controlling for possible confounders. The strong and moderate studies reported mixed outcomes, i.e. some found that cultural adaptation(s) were effective, while others did not find statistically significant effects. Hence, we could not find a clear association between quality of the studies and effectiveness.

### Overall effectiveness of interventions per behavior

Five out of 17 studies that tested one or more cultural adaptations found statistically significant results on the effectiveness of cultural adaptations. Regarding smoking cessation, three studies (of four) reported statistically significant decrease in smoking [16]–[18] and one study (of six) regarding diet and PA [26] demonstrated effectiveness on primary outcomes. No studies aimed at only diet or only PA reported significant effectiveness [19]–[24], [28], [33]. The study that was aimed at all three behaviors reported effectiveness on smoking cessation and energy expenditure [31], [32]. The interventions and the cultural adaptations that were tested (with or without effectiveness) are described below.

### Cultural adaptations that were reported to be effective

Table 3 shows the content of the five intervention studies with cultural adaptations that reported statistically significant effectiveness on primary outcomes. Three studies targeted smoking cessation [16]–[18] and one targeted diet and PA [26]. The study by Cene et al. [31], [32] which targeted smoking, diet and PA, reported effectiveness on smoking behavior and energy expenditure [31], [32]. All studies [16]–[18], [26], [32] incorporated a package of adaptations.

**Table 3:** Studies that reported effect on adaptations tested.

Reference	Description of intervention			Dose received		Effect
	Intervention group (adapted intervention)	Control group (basic intervention)		Intervention Group	Control Group	
***Smoking cessation***						
Wetter et al. 2007 [17]	Basic intervention plus 3 additional proactive counseling calls at 1, 2 and 4 weeks after the initial call. The counseling approach focuses on ‘practical counseling’ and on intra- and extra treatment social support. Motivational techniques were also included. The counseling was delivered in Spanish and tailored to Hispanic values such as culture of respect, pleasant and agreeable family and positive social relationships.	Single CIS telephone counseling call and offer of Spanish language self help materials (if preferred):		All calls: 83%; 3 of 4 calls: 92%	Single Call: 100%	Abstinence rates after 5 weeks: IG 20.3%: vs. CG 11.7%.
	**Adaptations tested**	- Guia Para Dejar de Fumar (Guide to Quit Smoking)				Abstinence rates after 12 weeks: IG 27.4% vs. CG 20.5%.
	*Deep-structure adaptations*	- Datos y Consejos Para Dejar de Fumar (Facts and Advice to Quit Smoking)				After controlling for demographic and tobacco related variables treatment effect significant (OR = 3.8; p: 0.048).
	- Additional proactive calls	- Usted Puede Dejar de Fumar (You Can Quit Smoking)				
	- Focus on practical counseling and social support	- Cancer Facts.				
	- Tailoring to Hispanic values (respecto, simpatico, familismo, personalismo)					
Orleans et al. 1998 [16]	A self help guide (pathways to freedom) targeted to African Americans (AA) (use AA models, use geared to smoking patterns of AA)	A copy of the NCI's generic quit smoking guide ‘Cleaning the Air’.		Reading guide: 58.2%.	Reading guide:54.7%	Abstinence rates at 6 months:
	Personal tailored counseling with special attention to motives and barriers more common among AA (*deep-structure adaptations*)	CIS telephone counseling by trained smoking cessation specialists used.				IG 74 (16.2%) vs. CG 62 (14.4%) (n.s.)
	**Adaptations tested**					12 months:
	*Surface-structure adaptations*					IG 36 (25%) vs. CG 18 (15.4%) (p<0.05) (Non-response coded as “still smoking”.)
	- Adaptation of self help guide					
	*Deep-structure adaptations*					
	- Incorporating values in self help guide					
	- Tailored counseling with special attention to motives and barriers more common among African Americans					
Woodruff et al. 2002 [18]	Three culturally adapted telephone calls in combination with four home visits in Spanish. Intervention was delivered by lay advisors (promotores) according to a curriculum in Spanish. All sessions were culturally adapted.	Standard telephone helpline in Spanish. Every caller received a six minute screening interview assessing smoking history, dependence, self efficacy, and readiness to quit. After first call, structured telephone sessions were initiated by a trained counselor in a proactive manner. It arranged follow-up sessions according to the probability of relapse. There were up to 6 calls.		Mean participation in sessions: 3.44 (sd 3.25)	Self reported: % called helpline, in last three months: 24%.	IG 20.5% vs. CG 8.7% (CO validated) (p <0.005)
	- Communication style and value congruent with Latino culture.					Including missing cases:
	- Intervention based on social cognitive principles, congruent with findings among Latino smokers.					IG 17.3% vs. CG: 8.3% (CO validated) (p<0.05)
	- Focus was on social and family concerns, rather than focus on individual.					
	- Intervention was based on cognitive principles and focus was on social and family concerns					
	**Adaptations tested**:					
	*Deep- and surface-structure*					
	- Home visits rather than telephone calls only					
	- Communication style,					
	- Using social cognitive principles congruent with Latino smokers					
	- Focus on family concerns					
***Diet and physical activity combined***						
Babamoto et al. 2009 [26]	Community health worker (CHW):	Case management	Standard care	CHW contacts in 6 month intervention on average (personal plus telephone)	Not reported	≥2 fruit/day increase
	Standard care plus highly trained CHW.	Standard care plus consultation with linguistically and culturally sensitive case manager (registered nurse). Case management involved individual sessions using standardized clinic protocols based on ADA recommendations, and meeting patients needs as needed (e.g. referral of community resources).	Standard care by physician and/or nurse practitioners: Standardized diabetic education materials in Spanish and English tailored for the local population provided at initial clinic visit.			At 6 month follow up: CHW 26% vs. Case management 28% vs. Standard 2% (p<0.05 between groups; p<0.05 within groups for CHW and Case management)
	CHWs were lay-people whohad diabetes or had experienced it through family or friend. They were paid clinical staff and underwent an extensive training course in diabetes standards but also in self-management strategies incorporating patient cultural and spiritual beliefs and health behavior change theory.					Increase ≥2 vegetable/day
	During 6 month intervention CHWs conducted tailored individual and family educational sessions based on ADA guidelines and culturally appropriate materials based on stages of change. CHWs also made follow-up phone calls to monitor progress.					At 6 months follow up: CHW 37% vs. Case manegement 25% vs. Standard care 8% (p<0.05 between groups; p<0.05 within groups for CHW and Case management)
	**Adaptations tested**:					Decrease ≥2 fatty foods/day:
	*Deep-structure adaptations*					At 6 month follow-up: CHW 13% vs. case management 2% vs. standard care 9% (p<0.05 within groups for CHW)
	- Cultural tailoring by CHW					Increase ≥3/week exercise:
	- Family educational sessions					CHW 35% vs. case management 13% vs. standard care 18% (p<0.05 between groups; p<0.05 within groups for CHW and standard care)
	- Use of culturally adapted materials					Decrease BMI:
						CHW 0,5 vs. case management 0,3 vs. standard care +0,3
***All three behaviors***						
Becker et al. 2005, Cene et al. 2008 [31], [32]	Consist of same elements and individual tailored to individual risk score.	Physical assessment, education, pharmacotherapy and adherence monitoring.		n.a.	n.a.	*Difference in decrease percentage smoking*
	Care took place at a nonclinical site in the community. Physical assessment, evaluation for pharmacotherapy and monitoring adherence done by nurse practitioner. Smoking cessation and exercise counseling by community health worker. All siblings received pharmacy card for free pharmacotherapy.	Individually tailored recommendations specific to the individual's risk factor status				IG 6% vs. CG 3%
	**Adaptations tested**	Risk information sent to primary care physician, who provided control patients with usual care, including office visits, education pharmacotherapy and adherence monitoring. Control patients were instructed to ask GP for pharmacy card for free pharmacotherapy.				Difference at post test: p<0.01.
	*Surface-structure adaptations*					*Decrease in percent energy from total fat*
	- Counseling in nonclinical site in community					IG 0,4 vs. CG 0,8 decrease
	- Counseling by community health worker					Difference at post test n.s
	- Easy access to free pharmacotherapy					*Decrease percent energy from sweets*
						IG 1,9% vs. CG: 1,9%
						Difference at post test: n.s.
						*Energy from expenditure, mJ/d*
						IG:+0,4 vs. CG -0,4, p<0.05
						*After 5 year results of smoking:*
						Significant increase in abstinence (IG 14% vs. CG 17%). No differences between the two groups

#### Smoking cessation

The content of the package of adaptations varied greatly. The adaptations were all added to standard telephone counseling that had been adapted for language and culture. Wetter et al. [17] provided proactive telephone calls in the adapted intervention. These focused on practical counseling and social support as well as Hispanic values like ‘culture of respect’ and ‘pleasant and agreeable family’ (deep-structure adaptations). The adapted intervention resulted in statistically significant higher abstinence rates after 12 weeks (27.4% vs. 20.5%) after controlling for demographic and tobacco related variables. Orleans et al. [16] adapted standard telephone counseling and a self help guide. Telephone counseling was tailored to motives and barriers specific for African Americans (deep-structure adaptations). The self help guide used African-American models and provided information about smoking among African Americans (surface-structure adaptations) as well as incorporating African-American values (deep-structure adaptations). At 12 months follow-up abstinence in intervention group was statistically significantly higher (25% vs. 15.4%). Woodruff et al. [18] replaced three of the six standard telephone consultations by four home visits. In the sessions the communication style was adapted and social cognitive principles were congruent with Latino smokers. In addition they focused on family concerns (deep- and surface-structure adaptations). At three months, seven day abstinence rates were statistically significantly higher in the intervention group (20.5% vs. 8.7%).

#### Three behaviors

In the study of Cene [32] the basic intervention consisted of consultations with a healthcare provider in a clinical setting and free pharmacotherapy. In the adapted intervention a community health worker (CHW) provided consultations in a non-clinical community setting (surface-structured adaptations). At one year follow-up smoking prevalence decreased significantly more in the intervention group (decrease of 6% vs. 3%). Energy expenditure increase was statistically significantly higher in the intervention group. Regarding the outcomes BMI, LDL cholesterol and metabolic equivalent [31], [32] no significant effects were found at one year follow-up.

#### Diet and PA

The intervention of Babamoto [26] had three intervention arms: 1) counseling by a healthcare provider, using Spanish-language materials culturally adapted to the local population; 2) counseling by healthcare provider plus a professional case manager (who used standardized clinical protocols, was trained to be culturally sensitive, and was able to communicate in Spanish) and written materials; and 3) an enhanced counseling intervention which consisted of the healthcare provider plus cultural tailoring by a culturally competent trained CHW, family educational sessions and the written materials (deep-structure adaptations). At six months follow-up statistically significantly more respondents reported increasing their fruit and vegetable intake in the CHW and the case manager group compared with the standard care group. Fat intake was statistically significantly lower in the CHW group only (13% decrease vs. 2% and 9% decrease respectively).

### Interventions with cultural adaptations that were reported not to be effective

Twelve of the 17 studies found no statistically significant effects of the cultural adaptation on primary outcomes (Table 4): one (of four) on smoking [15], five (of six) on diet and PA combined [25], [27]–[30], four (of four) on diet only [19]–[22], and two (of two) on PA only [23], [24]. Eight studies (of 12) tested the effects of one type of adaptation [19]–[22], [25], [27]–[30] and four a package of adaptations [15], [23], [24], [29].

**Table 4:** Description intervention and outcomes of studies that didn't report an effect of cultural adaptation.

Reference	Description of intervention			Dose received			Effect
	Intervention group (Adapted intervention)	Control group (Basic intervention)		Intervention group	Control group		
***Smoking cessation***							
Nollen et al. 2007 [15]	*Basic intervention with adaptation of video and self help guide: The Harlem Health Connection*'*s ‘Kick-It’* video incorporated core African-American cultural values (communalism, religion/spiritualism, connection to ancestors and history, storytelling).	4 weeks of 21 mg/day transdermal nicotine patches.		Read most or all of the written guide 172 (68.8%)	Read most or all of the written guide 149 (59.6%)		No sign. differences in 7-day abstinence at month 6 between the intervention and control groups:
	‘*Pathways To Freedom: Winning the Fight Against Tobacco’* addresses issues specific to African Americans (AA role models, stronger smoking norm, AA specific smoking pattern, AA targeted advertising, cultural and socioeconomic influences).	48-min videotape ‘How to quit’ and the American Lung Association's					IG 18.0% (45/250) vs. CG14.4% (36/250); *p = 0*.27).
	**Adaptations tested**	*‘Freedom From Smoking’* guide (designed for general population) containing lectures by specialists on nicotine control, nutrition and stress management.					*Secondary outcomes*: no significant differences
	*Surface-structure adaptations*	‘Freedom from smoking’ self help guide served as the take home reading material for the control patients.					- 7-day Abstinence at week 4: 25.6% in both groups
	- Adaptations in Self help guide						- Reduction in cigarettes per day week 4: IG: −9.44; CG-9.40
	*Deep-structure adaptations*						- Reduction in cigarettes per day, month 6: IG: -6.30; CG: −6.77
	- Cultural values, norms influences in self help guide						
	- Incorporation of cultural values in video						
***Diet and physical activity combined***							
Keyserling et al. 2002 [29]	A: Clinic (4) + community based (2 group sessions and monthly telephone contact with (lay) community DM advisor)	B: Clinic only –4 clinic visits	C: Minimal intervention	A	B		(kcal per day attributed to PA)
	Clinic counseling augmented with group sessions and telephone contact. Designed to address issues related to cultural translation (i.e. to make it relevant to the cultural context of participants).	Counseling based on tailored advice regarding diet and PA, focus on behavioral change strategies.	Participants received a number of written pamphlets regarding diabetes, healthy eating and PA	Group sessions:	Clinic attendance did not differ between groups A and B		Difference between groups:
	DM advisor is a non-professional peer advisor. Role is to provide social support and feedback, reinforce personal diet and activity goals and to assist with group sessions.	Simple language used in back-up written materials,		1: 28 (58%)			Group IG(A) vs. CG(C)
	Group sessions were designed to promote readiness to change and to provide social support. Topics included demonstration and taste testing of modified recipes, role modeling (within group)	Southern-style cookbook with low cost recipes.		2: 26 (49%)			6 months 36.2
	**Adaptations tested**	Tip sheets addressing general themes.		310 phone calls (9.7 per participant)			12 months: 52.0; p = 0.019
	*Deep- and surface-structure adaptations*			Group session 3: 35 (59%)			Group CG(C) vs. CG(B)
	- Community based group sessions including cultural adaptation to participants			261 phone calls			6 months 44.5; p = 0.036
	- Monthly telephone contact with lay community health advisor						12 months: 21.7; p = 0.31
							Group IG (A)vs. CG(B)
							6 months 8.3; p = 0.70
							12 months 30.4; p = 0.17
							*Other outcomes*
							Calories from saturated fat (%) and total energy intake per day: No significant differences between groups.
Ard et al. 2008 [25]	All African-American intervention groups	Mixed race intervention groups		Attending ≥17 group sessions: 48.3%	% Attending ≥17 group sessions: 38.7%		None of the differences between IG and CG were statistically significant.
	- Weekly sessions as in the control group	- 20 weekly group sessions led by an African-American interventionist trained on culturally appropriate delivery of the program.					*Primary outcome:*
	**Adaptation tested**	- Some key strategies: self-monitoring of diet and PA; reducing portion sizes; substituting alternative foods; modifying the original items to be lower in calories and fat; increasing Fruit and Vegetable (F&V), and fiber intake; increasing PA; identifying problematic situations and making specific action plans to deal with those situations; and developing core food choice competencies					Mean weight loss both groups: 4.2kg.
	*Surface-structure adaptations*						Mean BMI change −1.5 kg/m^2^
	- Group composition						*Secondary outcomes*
							F&V intake, fiber intake and % calories from fat did not differ between IG and CG
							Physical activity: In both groups the proportion who reported at least 180min moderate-vigorous PA per week increased
Fitzgibbon et al. 2005 [28]	Faith-based component added to the culturally tailored weight loss intervention as in the control group	Culturally tailored weight loss intervention		Attending ≥75% of the classes: 50%	Attending ≥75% of the classes: 52%		No statistically significant differences between IG and CG in change in BMI (difference IG-CG = −0.34 in weight (difference −0.95kg), fat consumption (difference −0.25 %kcal), and energy expenditure in PA (difference −0.25 kcal/kg/day)
	- Addressed in addition faith/spirituality issues in a structured and systematic manner (each week a scripture from the Bible was incorporated into the content of the intervention)	- Twice weekly meetings for 12 weeks: a weekly 90-minute meeting (interactive didactic component + exercise component) and a weekly 45-minute exercise session					IG and CG both exhibited some pre-post weight loss.
	- Delivered by a woman with experience conducting health risk reduction interventions with minority populations and knowledge of the Bible and scripture readings	- Teaching and supporting the use of tools (e.g. daily self-monitoring of food intake and physical activity, reinforcement, modeling, stimulus control and social support)					IG and CG both exhibited some pre-post lower fat consumption
	**Adaptation tested**	- The recruitment and intervention protocol emphasized cultural tailoring and cultural sensitivity *surface-structure* (e.g. people, places, language and locations devised to AA preferences) and *deep-structure* (e.g. respect for verbal communication skills, connection to ancestors and history, and commitment to family and other obligations)					Total energy expenditure increased only statistically significant in the control group
	*Deep-structure adaptations*	- Healthy ways of preparing traditional AA food, emphasized family and social support, offered childcare, discussed multiple family obligations and provided advice on how to prepare healthy food when serving a large extended family. Sharing medical “stories,” about health consequences of unhealthy eating and physical inactivity that involved well-known or historical figures.					
	- Faith-based component						
Staten et al. 2004 [30]	PC + HE + CHW (community health worker):	PC + HE (Health Education):	PC (provider counseling) only:	n/a	n/a		Intervention group had slightly higher fruit/vegetable intake at follow-up.
	Group received the same as the PC + HE group but also communicated on a regular basis – semi weekly to monthly with community health workers. The CHW provided information and support and organized bi-monthly walks.	In addition, this group was offered 2 health education classes and monthly newsletter for 12 months.	Participants received brochures and nurse practitioner discussed benefits of and barriers to increasing PA and Fruit and Vegetable consumption this advice was tailored to individual.				Change in (V&F) intake:
	CHWs also encouraged participants to find walking partners and support each other in health improvement goals.	HE classes based on social-cognitive theory	Tailored materials based on current behavior. PC based on patient-provider communication model.				IG: 0.26 vs. PC + HE: −0.23 vs.
	**Adaptations tested**						PC:−0.59
	*Deep- and surface-structure adaptation*						All groups increased their physical activity,
	- Contact with CHW						PC + HE en IG appeared to increase the vigour of their daily activity but the tool used to measure PA was not sensitive enough to quantify this parameter.
Campbell et al. 2004 [27]	Combined intervention (TPV + LHA):	Theory based training for the LHA to disseminate info and promote interactions and activities.	TPV: theory based material sent to individuals home.	Majority of participants reported having heard about cancer prevention in the past year; this was greatest in the TPV group (70%).			TPV intervention was most effective in increasing number of servings F&V: Combined = .3.7 vs. TPV = 3.9 vs. LHA = 3.5, (p = 0.02)
	Participants received TPV materials at home and had a LHA at the church.	LHA used same theoretical constructs as TPV but dispersed by LHA who would also support changes among church members (social support model).	Tailoring based on stage of change, social cognitive theory, health belief model. 4 personalized computer-tailored newsletters and four targeted videos mailed at 2,4,6,9 months after baseline. Newsletters tailored to appeal to African Americans, included church-specific messages, from the pastor and testimonials from church members – these were also featured in the videos made for each of the churches.				TPV was most effective in increasing % meeting 5-a-day recommendation for Fruit and: Vegetables Combined = 26.4% vs. TPV = 21.7%, vs. LHA = 15.4%, (p = 0.04)
	**Adaptations tested**						No differences between all groups in intake of % calories from fat and in recreational activity (MET hrs/week):
	*Surface-structure adaptations*						Combined = 9.7% vs. TPV = 9.7 vs. LHA = 10.6, or in meeting PA recommendations: Combined = 45.9%. vs. TPV = 46.3% vs. LHA = 43.9%.
	- Use of LHA						
***Diet***							
Kreuter et al. 2005^1^ [21], [34]	Behaviorally and culturally tailored magazines (BCT + CRT)	Culturally relevant magazines (CRT)	Behaviorally tailored magazines (BCT)	BCT+ CRT	BCT		Median F&V intake change score in the combined intervention was highest, although only significantly greater than that of the CRT, but not significantly greater than the BCT among all women
^1^ Control Group without basic intervention not reported in table.	- 6 Tailored magazines as in the BCT and CRT group, however consisting of culturally relevant stories as well as behaviorally concept stories as in the control groups (equally divided)	- 6 Tailored magazines as in the BCT group, however in CRT magazines stories were tailored on 2 of the 4 cultural constructs religiosity, collectivism, racial pride, and/or time orientation.	- 6 tailored magazines, 18-month period, these included 10 tailored stories. 6 addressing Fruit and Vegetable (F&V) intake, knowledge, self-efficacy, perceived barriers, perceived importance of eating more F&V, level of interest in eating more F&V, and actual dietary practices.	Attention:	Attention		Mean change of F&V intake:
	Magazines for all three conditions: the front cover featured artwork from local African-American (AA) artists. Graphics and text developed with participation from AA community.		4 on topics not related to either study outcome (tailored on participant characteristics and interests)	1-month: 3.73 (SD 1.5)	1-month: 3.78 (SD 1.38)		At 6 months: Combined group: +0.49 servings, vs BCT: −0.14 servings vs. CRT: +0.07 servings.
	**Adaptations tested**			6-month: 4.00 (SD 1.27)	6-month: 3.99 (SD 1.27)		At 18-month follow-up: mean change combined group +0.96 servings vs. BCT: +0.43 servings vs. CRT: +0.25 servings
	*Deep-structure*				CRT		F&V intake in the combined group was marginally more effective than other conditions among women with lower motivation at 6-month (p = .096) and 18-month (p = .003)
	- Adaptation of culturally relevant stories				Attention:		
					1-month 3.57 (SD 1.49)		
					6-month 3.88 (SD 1.31)		
Elder et al. 2005 [20], [33]	Promotora-tailored print material condition:	Tailored print condition	Usual care	A much larger percentage (29.3) of participants in the *promotora* condition returned all activity inserts; only 14.6% returned none.	In the tailored condition 9.7% returned all activity inserts; 41.9% returned none.		No significant differences were detected among groups for the primary outcomes % calories from fat and total dietary fiber.
	- Tailored print newsletters and activity insert as in the tailored print condition. In addition: weekly home visits or telephone calls from sequentially assigned promotoras over a 12-week period.	12 weeks of tailored print newsletters (based on baseline data) and activity inserts mailed to their homes					Percent calories from fat decreased: Promotora: 2.2% vs. tailored print: 0,6% vs. usual care: 1.5%
	- Promotoras were Spanish-language dominant, naturally empathetic, able to develop rapport and to be neutral and nonjudgmental, perceived as a role model in the community, and interested in helping women change lifestyle behaviors.	Materials provided feedback, opportunity for personalized goal setting, and for dealing with barriers.					Total dietary fiber decreased: Promotora: 1.1g vs. tailored print: 0g vs. usual care: 0.9g
	- Promotora's used the skills acquired in the program, as well as their natural ability to provide support (informational, instrumental, and emotional) and encouragement to negotiate behavioral change goals. Relied on the participant's weekly tailored newsletters to guide discussions and suggest opportunities for skill development.						Secundary outcomes:
	**Adaptations tested**						The promotora group was significantly or marginally lower than the control and tailored groups for energy, total fat, total saturated fat, and total carbohydrates (p.<.05–.10).
	*Surface-structure adaptation*						The promotora group was significantly lower than the tailored group for glucose and fructose (p<.05).
	- Use of Promtora's						For every outcome, the group differences seen were no longer significantly different at 6-month follow-up and 12-months follow-up.
							Instead, there were group-by-time interactions; effects achieved by the promotoras dissipated over the 12-month follow-up period while the effects of the tailored group concurrently improved.
Campbell et al. 1999 [19]	The spiritually oriented bulletin	The expert oriented bulletin:		72.9% recalled receiving a bulletin	64.6% recalled receiving a bulletin		F &V consumption (servings per day) increase: Spiritual: 0.6 vs. expert: 0.7
	- received a bulletin like in the expert oriented condition, however with religious language and messages from the church pastor	Received a bulletin that used scientific language and messages from nutritionists					The IG group consumption did not differ significantly from the CG, controlling for baseline consumption, education, age, and study effects
	- Bulletins began with a message “From the pastor's desk” that was written by the pastor of the participant's church and included a photograph of the pastor, and ended with a “Five-a-Day-Grace” written by the pastor	- These bulletins began with a “Welcome to Five-a-Day” message that stated that the bulletin was based on “the latest research” and ended with a “Featured Fruit” message					
	- Tailored messages about perceived social support and the top three perceived barriers to eating Fruit and Vegetable (F&V) were written with spiritual language and biblical allusions	- Included a.o:					
	- Church-oriented artwork created by an African-American community member was included	- Tailored messages about perceived social support and the top three perceived barriers to eating F&V were written with references to nutrition research and medicine					
	- An article “why God wants you to eat healthy” was also included	- Artwork that depicted a family rather than a church scene by the same AA community member					
	- A bookmark was attached to the back of the bulleting that featured a passage from the Book of Genesis about F&V	- Each bulletin featured personalized messages tailored on the basis of baseline information.					
	**Adaptations tested**						
	*Deep-structure adaptations*						
	- Spiritually orientation of health message						
Rescinow et al. 2009 [22]	Newsletters targeted at ethnic identity	General targeted newsletters for a Black-American audience with a slight Untailored, ethnically neutral graphics accomplished by generally featuring images without people or other racial or ethnic cues. Slight ethnocentric focus.		61% reported receiving 3 newsletters (25%, 2)	66% reported receiving 3 newsletters (26%, 2)		Increase in daily mean Fruit and vegetable (F&V) intake:
	- Newsletters as in the control group, but targeted at one of the 16 ethnic identities (the 16 types were based on various combinations of five core types: assimilated, Black Americans, Afro centric, Bicultural, and Multicultural, with cultural mistrust as subtypes of the Black American and Afro centric identity types). E.g. risks of Black Americans on chronic diseases were specified for participants with a Black American EI.	- 3 Newsletters by mail (once a month)					IG: 1.1 servings per day vs. CG: 0.8 servings per dag. (ns.)
	- Graphics depicting eating and social scenes were used to tailor the graphics by EI type, gender, age, marital status and children living in the home. E.g. the Black American newsletters featured images of almost exclusively Black Americans, while the assimilated newsletters primarily featured images of individuals of other cultures with ethnically indistinct clothing, hair, and jewellery. Participants with a dual Afro centric EI type received Afro centric images mixed with images from their second EI type, etc. All other factors of the photos were constant.	- Each newsletter included two recipe cards accompanied by small bags of spices and either a magnetized refrigerator notepad or a magnet with Fruit and Vegetable (F&V) serving size information					Only among the subpopulation with an Afro -centric identity a significantly larger increase in F&V intake was reported:
	**Adaptations tested**	- Text contained intermittent use of the participant's name and tailoring on F&V intake, sociobehavioral variables (e.g. preferences, dietary limitations, social roles for shopping and cooking, barriers, outcome expectations, and demographics)					IG: 1.4 servings per day vs. CG: 0.43 servings per day; p <0.05.
	*Deep-structure adaptations*	- Draft newsletter text, layout and messages were tested and refined by African Americans and, among others, experts in Black identity theory.					
	- Information targeted at ethnic identity	Target reading level was approximately sixth grade.					
***Physical activity***							
Chiang et al. 2009 [23]	Similar 8-week walking program plus culturally sensitive monitor by phone every week including:	8-week walking program, all materials translated to Chinese.		Not reported	Not reported		Duration of walking was not significantly different between the IG and CG. No figures about effect available.
	- Emphasize Chinese cultural value of authority (opinions of people whom they respect).	Monitor by phone every week					
	- Family member involvement (signing informed consent by one of family members).						
	- Harmony and balance (harmony with the natural environment, social environment, and family is a way of life and will promote health and prevent illness)						
	**Adaptations tested**						
	*Deep-structure adaptations*						
	- Incorporating cultural values						
	- Involvement family						
Newton et al. 2004 [24]	Culturally Sensitive Exercise Counseling (CS), identical to SB except for 4 culture specific key elements (surface- & deep-structure):	Control group 2:	Control group 1:	Rate of attendance: 61%	55%	73%	Both the CS and the SB group demonstrated a significant effect on the primary outcome measure cardio respiratory fitness.
	1) all group members were African-American (AA)	Standard Behavioral Exercise Counseling (SB). 10 group intervention sessions over 6 months. weekly during month 1, biweekly during month 2 to 3 and monthly during months 4 to 6	Physician Advise (PA), a minimal treatment corresponding to the exercise guidelines that a healthcare provider would typically give to a sedentary individual.	(Not significant different)			VO_2_ max. change (ml/kg/min):
	2) the sessions were led by AA						IG: 0.45 vs. CG2: 1.44 vs. CG1: −0.88
	3) sessions were conducted at site located in the AA community						No significant differences in number of days per week of exercise between the groups at post treatment.
	4) materials were designed to address socio-cultural concerns of AA regarding exercise (beliefs, worldview & history, depictions of AA)						Exercise change (days/week):
	**Adaptations tested**						IG: 2.75 vs. CG2: 3.10 vs. CG1: 2.00
	*Surface-structure adaptations*						CS- and SB-group increased minutes per week of walking from baseline to 6 months compared with PA group.
	- Only African-American group members and counselors						
	- Location						
	*Deep-structure*						
	- Incorporating socio cultural values and cultural values regarding exercising						

#### Smoking cessation

One study evaluated the cultural adaptation of a self help guide and videotaped education sessions designed for the general population [15]. Cultural adaptations consisted of surface-structure adaptations such as the use of African-American role models and deep-structure adaptations by incorporating socio-cultural values into the self help guide and video. Smoking cessation rates, although lower in the intervention group, did not differ significantly between groups.

#### Diet and PA combined

One study [29] compared standard clinic-based counseling which already included some culturally specific written materials with an intervention condition that employed group sessions and monthly telephone contact with a lay health advisor as additional adaptations. Both the group sessions and contact with the lay health advisor were adapted to the socio-cultural values of the target group (deep- and surface-structure adaptations). This study was not sufficiently powered to demonstrate differences between the two conditions.

Four studies investigated a single adaptation. One study [25] investigated whether group composition (ethnically mixed versus homogenous groups) in a group intervention influenced weight loss among African Americans (surface-structure adaptation) and found no difference between the groups. Fitzgibbon et al. [28] evaluated incorporation of faith in a weight loss intervention. This deep-structure cultural adaptation showed no significant effectiveness. In the study of Staten et al. [30] the cultural adaptation consisted of extra contacts with a CHW who communicated with patients, provided information, organized group walks and encouraged participants to find walking partners (deep- and surface-structure adaptations). There were no statistically significant differences between groups at follow-up although the intervention group had a slightly higher fruit intake. Finally, Campbell et al. [27] tested the effectiveness of employing a lay health advisor (LHA) (surface-structure adaptation) in addition to tailored printed materials (TPV): in the intervention group the LHA provided the same information to respondents as they received at home, in the form of TPV. In this study the ‘TPV only’ condition had a statistically significant effect on fruit intake and percentage that meet the recommendations of five servings a day; implying that the cultural adaptation was not effective.

#### Diet

The studies of interventions aimed to change diet all assessed one type of cultural adaptation instead of a package of different cultural adaptations. Kreuter et al. [34] studied the effect of cultural tailoring and behavioral tailoring using magazines (deep-structure adaptations). At six months follow-up African-American women receiving a combination of both behaviorally tailored as well as culturally tailored magazines had greater increases in daily fruit and vegetable consumption although this was not statistically significantly different from the group receiving behaviorally tailored magazines only. Elder et al. [20], [33] assessed the effect of weekly home visits and/or telephone calls from LHA in addition to tailored print materials. LHA provided the same information as the print materials (surface-structured adaptation). No effects on primary outcomes were found at 12 weeks and effects on other outcomes disappeared at six-month follow-up. The study by Campbell et al. [19] tested whether a behaviorally tailored bulletin incorporating health messages with a spiritual orientation (deep-structure adaptations) were more effective when the source was the pastor versus an expert (surface-structure adaptation). No differences on fruit and vegetable consumption were found between the two conditions. Finally, Rescinow et al. [22] tested the effectiveness of targeting messages to the ethnic identity of respondents by using untailored, ethnically neutral graphics versus newsletters with information targeted at ethnic identity in a study population of African Americans. A significant increase in fruit and vegetable intake was only found for a subpopulation of participants with Afro-centric identity but not for the total group.

#### PA

The two culturally adapted interventions aimed at increasing PA without treatment effect tested a package of cultural adaptations. Chiang et al. [23] investigated the incorporating of cultural values in the messages and the involvement of family members in a standard eight-week walking program with translated written materials for Chinese Americans and weekly telephone monitoring. At follow-up walking duration was not significantly different between the two study arms. In the study of Newton et al. [24], deep- and surface-structure adaptations were made to an intervention consisting of ten group sessions. In the adapted intervention, all group members and the counselor were African American, the location was preferred by the target population and socio-cultural values regarding exercising were incorporated. There were no differences in cardio respiratory fitness and exercise frequency between the adapted and basic intervention group.

### Are there features of the adaptations that may explain their effectiveness?

We could distinguish five broad categories of adaptations: level of adaptation, i.e. surface- vs. deep-structure; cultural values vs. interventions involving community health workers or lay health advisors; incorporating family vs. religious values; interventions employing intensive vs. non-intensive strategies; and use of a package of adaptations vs. one type of adaptation (Table 5).

**Table 5:** Features of the cultural adaptations.

	Cultural adaptation			Cultural values vs. lay health advisors		Categories of cultural values adaptations			Cultural adaptation imply higher intensity		Number of adaptations tested	
	Surface-structure	Deep-structure	Both	Cultural values	Involvement cultural health advisors	Religious values	Family values/family involved	Cultural values not further specified	Yes	No	Package	One
**Effective studies**												
*Smoking cessation*												
Wetter et al. [17]		x		x			x		x		x	
Orleans et al. [16]			x	x				x	x		x	
Woodruff et al. [18]			x		x		x		x		x	
*Diet and PA*												
Babamoto et al. [26]		x			x		x		x		x	
*All three behaviors*												
Cene et al./Becker et al. [31], [32]	x				x				x		x	
**Studies not reporting effect of adaptation**												
*Smoking cessation*												
Nollen et al. [15]			x	x		x				x	x	
*Diet and PA*												
Keyserling et al. [29]			x		x			x	x		x	
Ard et al. [25]	x				x					x		x
Fitzgibbon et al. [28]		x		x		x				x		x
Staten et al. [30]			x		x				x			x
Campbell et al. [27]	x				x	x			x			x
*Diet only*												
Kreuter et al. [21]		x		x		x				x		x
Elder et al. [20]	x				x				x			x
Campbell et al. [19]		x		x		x				x		x
Resnicow et al. [22]		x		x				x		x		x
*PA only*												
Chiang et al. [23]		x			x		x			x	x	
Newton et al. [24]			x	x				x		x	x	

#### Surface- vs. deep-structure adaptations

We found no indication that the level of adaptations influenced effectiveness. Studies that demonstrated statistically significant effects (n = 4) as well as several studies that did not (n = 9) included deep-structure adaptations. The same applies to surface-structure adaptations.

#### Adaptations based mainly on cultural values vs. involvement of lay health advisors/community health workers

We also observed no pattern of effectiveness when we distinguished the studies on the basis of adaptations that involved community health workers or lay health advisors versus adaptations mainly based on incorporating cultural values into intervention materials or in the counseling conducted by professionals.

#### Distinguishing incorporating family versus religious values

We found that we could distinguish topics used in the cultural adaptations, i.e. religious values, family values and/or family involvement and other cultural values which were not further specified. Statistically significant effects on primary outcomes were found by three interventions (of four) that incorporated family values and/or involved family members and by none interventions (of five) that incorporated religious values.

#### Intensity of the adaptation

In 9 of the 17 studies, the cultural adaptation implied an increase of the intervention's intensity (e.g. extra sessions with a lay health advisor). This was the case in all studies that reported statistically significant effects on primary outcomes.

#### Number of adaptations tested

Five studies (of nine) that incorporated a package of adaptations, e.g.; additional proactive calls together with tailoring to cultural values [17] reported statistically significant effects. Studies using one type of adaptation, e.g. use of homogeneous groups, [25] didn't show statistically significant effects (n =  eight).

## Discussion

To our knowledge, this is the first review that investigated the effectiveness of specific cultural adaptations in interventions for ethnic minorities targeted at smoking cessation, diet, and/or increasing PA. We identified 17 studies that evaluated the effectiveness of one or more cultural adaptations. The adaptations tested ranged from incorporating socio-cultural values of the target population to involving community health workers or lay health advisors; from employing a single adaptation to a package of adaptations; and from using deep-structure adaptations to surface-structure adaptations. In five studies the adapted intervention had a positive statistically significant effect on the primary outcomes. These were mainly interventions that targeted smoking cessation. Twelve studies showed no statistically significant effects on primary outcomes, although some studies presented trends favorable for cultural adaptations. We observed that interventions incorporating a package of cultural adaptations, cultural adaptations that implied a higher intensity and those incorporating family values were more likely to report statistically significant effects.

### Limitations

Before interpreting the results, the limitations of our review need to be considered. Like in all systematic reviews, publication bias (e.g. because studies were small or non-effective) may have limited the number of studies to be found. However, as our search did yield several small non-effective studies, we do not expect that this would have changed our results.

Second, the studies show heterogeneity in design and quality. Outcome measures varied too much to conduct a detailed analysis of effect sizes. In addition, follow-up time varied between studies. It may be expected that studies with a short follow-up interval would be more likely to report effects; however we did not find such a pattern. A similar argumentation can be followed for the quality of the studies; studies that have a strong design are less likely to be prone to bias compared with those studies with a moderate or weak quality score. For example, it may be that studies with a moderate or weak design have hidden effects due to a more motivated control group, or, in contrast, that effects were found just because of differences between the experimental and control group. In our analysis we found both moderate and strong designs in studies either with or without effect. Based on these results we conclude that we found no association with quality of the design and expect that the conclusions of our review are not likely to be affected by differences in design quality. If we consider the isssue of randomization as a separate study design issue, we see that two of the studies were not an RCT. Both of these studies showed no effect. It may be that some of the potential effects could be hidden due to bias in these studies. However, the fact that 10 out of the 15 RCT's also found no effect, means that this is unlikely to affect our conclusions.

### Interpretation of results

We found that studies that reported effects had tested a whole package of adaptations. Furthermore, the adaptations were more likely to be infused through the intervention as a whole and at several levels. For example, alongside the individual, the level of the family was included. Thus, in line with evidence about behavioral interventions in general, it seems that interventions targeting multiple levels result in larger effect than interventions focused at a single level [35].

The finding that including cultural adaptations is likely to result in a more intense intervention may reflect that enhancement of the intervention is an important aspect of the cultural adaptations. Most study populations in studies incorporating cultural adaptations have lower educational levels and are living in disadvantaged circumstances (e.g. [17], [21], [29]). It's possible that more time and attention is needed to make the information understandable to these groups [36] and that the effects of the included studies are also attributable to the adaptation to lower health literacy. This implies that adaptations to characteristics other than norms and values should also be taken into account when developing interventions.

One striking result of our review is that the effectiveness of specific cultural adaptations was seen mainly in studies on smoking cessation and less often in studies on diet and PA. Regarding PA, this is in line with an other review [37]. An additional factor which may play a role is the relationship between certain behaviors (like diet) and deeply rooted cultural values and personal identity. These behaviors may therefore be less changeable, as suggested by several studies [38]–[40]. As a result, changing these habits – including what, when, and how food is eaten – may need more attention when adapting an intervention.

### Implications for future research

The current review indicates that the current knowledge about the effectiveness of cultural adaptations is limited. To date most studies have been conducted in the United States among African Americans and Hispanics, which may not be generalizable to other population groups or to other countries. Therefore, future research needs to be conducted among other ethnic minority groups with different migration histories or local circumstances.

In addition the results indicate that the questions “what is a cultural adaptation?” and “how is it employed?” are important issues to consider at the inception of culturally sensitive interventions and their subsequent reporting. We started with the assumption that we would find adaptations such as the use of prevalence rates of diseases from the target population or the use of cultural values in the health promoting message. But, we found that factors as the intensity and of combination of adaptations appear to play an additional, important role. Our analysis was limited by its reliance on the authors' varying descriptions of the adaptations they applied. It seems that in order to move this field forward, more explicit description and discussion of these issues is needed.

The results of this review provide useful input for further research. We recommend, first, that the design of future studies should include a standard care arm with regular care as provided by general population without any extra interventions. Examples can be seen in Babamoto et al., Keyserling et al., and Newton et al. [22], [24], [29]. With this design we are better able to assess the effect of the basic intervention and the adaptations tested.

Second, although a broad mix of cultural components appears most effective, it remains important to identify the specific cultural adaptations that are most effective within this mix. This calls for more ‘experimental’ designs in which well-defined adaptations or combinations of adaptations are tested. Examples of studies that tested one type of adaptation are studies done by Resnicow et al. [22] on assessing the incorporation of ethnic identity and by Ard et al. [25] on assessing the effect of group composition. However, in these studies the basic intervention was already culturally adapted which may have limited the potential effect of the single adaptations. To assess the potential effect of single adaptations, studies can be designed similar to pharmacological trials. Adaptations that are considered for use could be tested in first- or second-phase studies solely and combined [41]. If effective, they could subsequently be included in larger controlled trials to provide insight into the package of adaptations that is most effective.

Finally, there are other characteristics of cultural adaptations that might be worth studying in more detail. The process of developing the cultural adaptation (e.g. was the target population involved in this development?), the way the adaptation was executed (e.g. related to the competence of the executers), the types of determinants addressed by the adaptations (e.g. enabling or cognitive factors) and the environmental levels addressed by the intervention (e.g. individual versus family level) are all examples of factors that may affect the effectiveness of cultural adaptations. More insight into these characteristics of cultural adaptations might help to select whether or not a cultural adaptation will contribute to the effectiveness of an intervention.

## Conclusion

The results of our review indicate that: 1) Culturally targeted interventions may be more effective if cultural adaptations are implemented as a package of adaptations, the adaptation addresses family influences, and where the adaptation implies a higher intensity of the intervention; 2) Adaptations in smoking cessation interventions seem to be more likely to be effective than adaptations in interventions aimed at diet and PA; 3) More systematic experiments are needed in which the aim is to gain insight in the best mix of cultural adaptations among diverse populations in various settings, particularly outside the US.

## Supporting Information

Checklist S1 **PRISMA Checklist.**
(DOC)
